# Identification of nucleoid associated proteins (NAPs) under oxidative stress in *Staphylococcus aureus*

**DOI:** 10.1186/s12866-017-1114-3

**Published:** 2017-10-02

**Authors:** Yuri Ushijima, Ryosuke L. Ohniwa, Kazuya Morikawa

**Affiliations:** 1 Graduate School of Comprehensive Human Sciences, University of Tsukuba, 1-1-1 Tennodai, Tsukuba, 305-8575 Japan; 2 Faculty of Medicine, University of Tsukuba, 1-1-1 Tennodai, Tsukuba, 305-8575 Japan; 3 Center for Biotechnology, National Taiwan University, Taipei 10617, Taiwan, Republic of China; 4 Present address: Department of Emerging Infectious Diseases, Institute of Tropical Medicine, Nagasaki University, 1-12-4 Sakamoto, Nagasaki, 852-8523 Japan

**Keywords:** *Staphylococcus aureus*, Nucleoid-associated proteins, Oxidative stress

## Abstract

**Background:**

Bacterial nucleoid consists of genome DNA, RNA, and hundreds of nucleoid-associated proteins (NAPs). *Escherichia coli* nucleoid is compacted towards the stationary phase, replacing most log-phase NAPs with the major stationary-phase nucleoid protein, Dps. In contrast, *Staphylococcus aureus* nucleoid sustains the fiber structures throughout the growth. Instead, the Dps homologue, MrgA, expresses under oxidative stress conditions to clump the nucleoid, but the composition of the clumped nucleoid was elusive.

**Results:**

The staphylococcal nucleoid under oxidative stress was isolated by sucrose gradient centrifugation, and the proteins were analyzed by liquid chromatography-mass spectrometry/mass spectrometry (LC-MS/MS). We identified 299 proteins in the nucleoid under oxidative stress, including 113 csNAPs (contaminant-subtracted NAPs). Comparison with the previously identified csNAPs in log- and stationary phase indicated that one fifth of the csNAPs under oxidative stress were the constitutive nucleoid components; importantly, several factors including HU, SarA, FabZ, and ribosomes were sustained under oxidative stress. Some factors (e.g. SA1663 and SA0092/SA0093) with unknown functions were included in the csNAPs list specifically under oxidative stress condition.

**Conclusion:**

Nucleoid constitutively holds Hu, SarA, FabG, and ribosomal proteins even under the oxidative stress, reflecting the active functions of the clumped nucleoid, unlikely to the dormant *E. coli* nucleoid compacted in the stationary phase or starvation.

**Electronic supplementary material:**

The online version of this article (10.1186/s12866-017-1114-3) contains supplementary material, which is available to authorized users.

## Background

*Staphylococcus aureus* is a Gram-positive bacterium that asymptomatically inhabits in the human/livestock nasal cavity and on skin surfaces [1]. It is also a major opportunistic pathogen responsible for a broad spectrum of infections ranging from superficial skin abscesses to more severe life-threatening diseases such as pneumonia, sepsis and toxic shock syndrome [2]. Hospital-acquired infections [2] as well as the recently highlighted community-acquired infections [3] are serious problems in clinical settings, largely because of the difficulty in the treatment with antibiotics due to the resistant strains, such as highly disseminated methicillin resistant *S. aureus* (MRSA) [4].

*S. aureus* has to cope with a variety of stresses in host environments [5, 6]. In commensal state, *S. aureus* relies on its resistance against lysozyme that is abundant in the nasal cavity [7, 8]. The prominent ability to survive under desiccation and hyperosmolarity helps its commensal growth or long-term survival on host or abiotic surfaces [9–11]. Once *S. aureus* invades into the host, it encounters the innate immune system including phagocytic cells such as neutrophil and macrophages. Reactive oxygen species (ROS) is the important bactericidal factor in the phagosome [12–14]. Superoxide anion is generated from oxygen by the membrane enzyme NADH oxidase [15]. Superoxide dismutase (SOD) catalyzes the conversion of superoxide anion into hydrogen peroxide [16, 17]. Ferrous iron catalyzes “Fenton reaction” that converts the hydrogen peroxide into the highly reactive hydroxyl radical [18, 19].

*S. aureus* can survive in phagosome for 3-5days [20], where the staphylococcal antioxidant enzymes responsible for the detoxification of ROSs must play critical roles. The anti-oxidant enzymes include SOD [21, 22], catalase that converts hydrogen peroxide into H_2_O and O_2_ [23], and the metallo regulon gene A (MrgA) [24]. MrgA belongs to the Dps protein family, and has both ferroxidase and DNA-binding/nucleoid clumping activities. Ferroxidase contributes to the oxidative stress resistance by reducing the concentration of ferrous iron that is required for the Fenton reaction [25]. Our mutational analysis of the ferroxidase center in MrgA suggested that the intact ferroxidase activity is essential for the oxidative stress resistance [24], while the nucleoid clumping by itself does not contribute to the resistance to the hydrogen peroxide stress [26]. The physiological significance of the nucleoid clumping is still unclear, but *S. aureus* is able to survive or proliferate under oxidative stress with its nucleoid clumped.

Previously, we comprehensively analyzed nucleoid-fraction proteins in four bacterial species including *S. aureus* and *E. coli* in the distinct growth phases, and identified contaminant-subtracted proteins enriched in the nucleoid fractions (csNAPs) [27]. Analyses of csNAPs suggested that the nucleoid components dynamically change from log phase to stationary phase. We also found that csNAPs contained global regulators, fatty acid synthesis enzymes, and oxidoreductases irrespective of the species and growth phases. In *E. coli*, the change in csNAPs towards the stationary phase was more drastic than in *S. aureus*. *E. coli* nucleoid undergoes compaction towards the stationary phase [28], and Azam et al. previously showed that major NAPs abundant in log phase cells (Hu, Fis, and Hfq) are replaced by Dps in the stationary phase [29]. Thus, the structural change in the *E. coli* nucleoid is associated with the drastic change in the major NAPs as well as other csNAPs. On the other hand, the NAPs composition of the clumped staphylococcal nucleoid under oxidative stress was elusive. Here, we aimed to clarify *S. aureus* csNAPs under the oxidative stress, and identified 113 csNAPs, one fifth of which were the constitutive nucleoid components irrespective of the oxidative stress. The characteristics of staphylococcal physiology will be discussed in terms of the csNAPs.

## Results

### Identification of *S. aureus* NAPs under oxidative stress

**Fig. 1:**
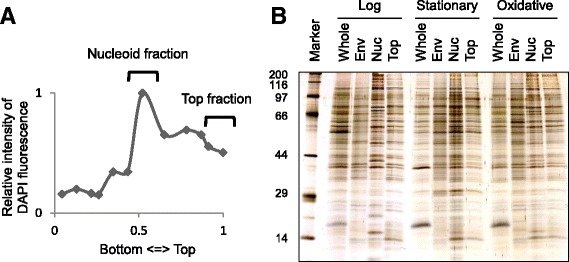
**a** The relative DNA amount under oxidative stress in the sucrose gradient detected by DAPI fluorescence. **b** SDS-PAGE of the whole cell lysate (Whole), the envelope fraction (Env), the nucleoid fraction (Nuc), and the top fraction of the sucrose gradient (Top). The proteins were visualized by silver staining. Sizes of the molecular weight markers are indicated in kDa on the left

The log phase *S. aureus* cells were exposed to the oxidative stress by the addition of 20 μM 9, 10-phenanthrenequinone (PQ) and cultivated for 30 min. The PQ produces oxidative stress [30, 31] that is sensed by PerR, leading to the MrgA induction and the nucleoid clumping [32]. Nucleoid isolation by sucrose gradient centrifugation, identification of the proteins by LC-MS/MS, and the determination of csNAPs were carried out as previously described [27]. DNA concentrations of the fractions in the sucrose gradient were measured (Fig. 1a), and the one with the highest DNA content was submitted to the analysis by LC-MS/MS. We also isolated envelope and top (soluble) fraction under oxidative stress, and analyzed them by LC-MS/MS.

Figure 1b shows the protein patterns of each fraction in the log phase, stationary phase, or under the oxidative stress. The protein profile of the nucleoid fraction under oxidative stress was similar to that in the log phase, but at least several signal intensities were different.

**Fig. 2:**
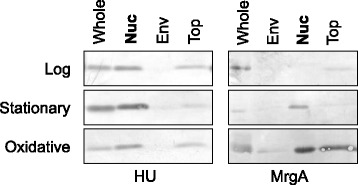
Western blot against HU (left panel) and MrgA (right panel). Five (HU) or 1 (MrgA) micrograms of proteins from the whole cell lysate, the envelope fraction, the nucleoid fraction, and the top fraction were separated by SDS-PAGE, and submitted to the Western blotting. The images of Log phase and Stationary (HU) are from Ohniwa RL et al., 2011 [27]

In *S. aureus*, heat-unstable nucleoid protein (HU) and metallo regulated gene A (MrgA) are proteins that locate in the nucleoid. HU is conserved among bacterial kingdom [28] and MrgA is an oxidative stress responsive nucleoid-clumping factor [32]. We examined the presence of HU and MrgA in each fraction by Western blot analysis (Fig. 2). HU localized in the nucleoid in all conditions (Fig. 2, HU). In the case of MrgA, the strong signal was detected in the nucleoid fraction under oxidative stress, and a comparable signal was also detected in the top fraction (Fig. 2, MrgA).

The numbers of proteins identified by LC-MS/MS were summarized in Table 1 (for comparison, the numbers of proteins in the log phase and stationary phase identified in our previous study were also shown [27]). We identified 299 proteins in the nucleoid fraction under the oxidative stress (Table 1, Nucleoid fraction). Here, HU was indeed detected in the nucleoid fraction (Additional file 1: Table S1, SA1305 DBH emPAI nucleoid 0.42), but MrgA was not. The latter was unexpected, because PQ-treated cells contain 30,000 MrgA molecules per cells [26], and the MrgA was enriched in the nucleoid fraction (Fig. 2). The reason for the absence of MrgA is unknown.

**Table 1:** Number of identified proteins

	Number of proteins identified			
	Nucleoid fraction	Envelope fraction	Top fraction	csNAPs
Log phase	225	274	150	92
Stationary phase	392	245	222	141
Oxidative stress	299	194	157	113

The number of Log phase and Stationary phase are from Ohniwa RL et al., 2011 [26]

In addition to HU, twenty-three proteins with high scores in the DNA/RNA binding prediction were identified [27] (Table 2). Six of them were transcription factors (*sarV*, *sarA*, *ctsR*, *rot*, *warR*, *msrR*). The proteins related to translation (*tsf*, *infC*, *infB* etc.), replication (*recA*, *dnlJ*, *top1*, *parE*, *parC* etc.), and inhibition of transcription termination (*nasG*), were also included.

**Table 2:** List of proteins identified in nucleoid fractions and predicted to be DNA/RNA binding proteins

Category	Phase	Genes
Transcription factors	Log phase	*codY, graR,* *rex* *,* *rot* *,* *sarA* *,* *sarH1* *,* *sarR* *,* *spxA* *, srrA,* *vraR*
	Stationary phase	*ahrC* *, codY,* *graR* *,* *mgrA* *,* *nreC* *,* *pyrR* *, rocA,* *saeR* *,* *sarA* *,* *sarR* *,* *sarH1* *,* *sarV* *,* *sarZ* *, srrA,* *tcaR* *, vraR, vicR*
	Oxidative stress	*sarV* *,* *sarA* *,* *ctsR* *,* *rot* *, warR,* *msrR*
Proteins involved in transcription, translation, replication, and DNA repair	Log phase	*fus,* *efp* *, tsf, tufA, end4, ermA,* *infA* *, nusG, pnpA, recA,* *rnc* *,* *rnh3* *, rpoA, rpoB, rpoC,* *rpoE* *,* *uvrC* *, xerD*
	Stationary phase	*lig, dnaN, fus,* *efp* *, tsf, tufA, gyrB, hsdR,* *infA* *, infB, infC,* *mfd* *,* *nusG* *, parC,* *parE* *, pnpA,* *rnc* *,* *rnj1* *, rnj2, rpoA, rpoB, rpoC,* *rpoE* *, gidB,* *ruvA* *,* *ssb* *,* *topA* *, Y1885*
	Oxidative stress	*tuf,* *infC* *, ftsZ,* *gpsB* *,* *rpoZ* *, nusG, rpoB, infB, recA, deaD, rpoD,* *sepF* *, top1,* *rnhB* *,* *dnlJ* *, parE,* *parC*

*E. coli* major NAP, HU was eliminated from the list.

Underline: csNAPs

The number of Log phase and Stationary phase are from Ohniwa RL et al., 2011 [26]

### List of contaminant-subtracted NAPs (csNAPs)

The isolated nucleoids must partly contain envelope and cytosolic proteins [27]. As described previously, we created the lists of contaminant-subtracted NAPs (csNAPs) by subtracting proteins that were abundant in the envelope or the top fractions from lists of NAPs [27]. Namely, csNAPs are defined as “Proteins detected only in the nucleoid fraction” plus “Proteins calculated to be relatively abundant in the nucleoid fraction”. The list of csNAPs in the log phase (csNAPs-log) and the stationary phase (csNAPs-st) can be found as Table S12 and Table S14, respectively in our previous report [27].

In the present study, 113 proteins were selected as csNAPs under the oxidative stress (termed csNAPs-ox) (Additional file 1: Table S2). Major surface proteins and cytosolic proteins (*clfB, spa, atpA, atpD, catA* etc.) in the list of NAPs were eliminated by this procedure, and not included in the list of csNAPs.

Comparisons of csNAPs-ox with csNAPs-log and csNAPs-st [27] are shown in Table 3. The 21.2% (24 proteins) and 25.7% (29 proteins) of csNAPs-ox was common in csNAPs-log and csNAPs-st, respectively. Thus, at least one-fifth of csNAPs are sustained regardless of the oxidative stress, suggesting that nucleoid protein does not completely exchange upon the oxidative stress. Regarding the global regulators, HU (*hu*) and SarA (*sarA*) were commonly found in the three csNAPs lists. Rot (*rot*) was shared by csNAP-log. Oxidoreductases such as alkyl hydroperoxide reductase (*ahpC*), alcohol dehydrogenase (*adh1*), and GMP reductase (*guaC*) were shared by csNAP-log. Fatty acid enzymes, FabZ (*fabZ*) and FabG (*fabG*), were common csNAPs among three conditions. In addition, some of the proteins related to transcription, translation, replication, and DNA repair were shared by csNAP-log or csNAP-st. Notably, the nucleoid in each condition contained ribosomal proteins with high emPAI values. Total numbers of ribosomal proteins identified as csNAPs or NAPs were similar among oxidative stress (35), log phase (43), and stationary phase (46) conditions (Additional file 1: Table S3), suggesting that ribosomes are sustained as the major parts of the nucleoid regardless of the oxidative stress.

**Table 3:**
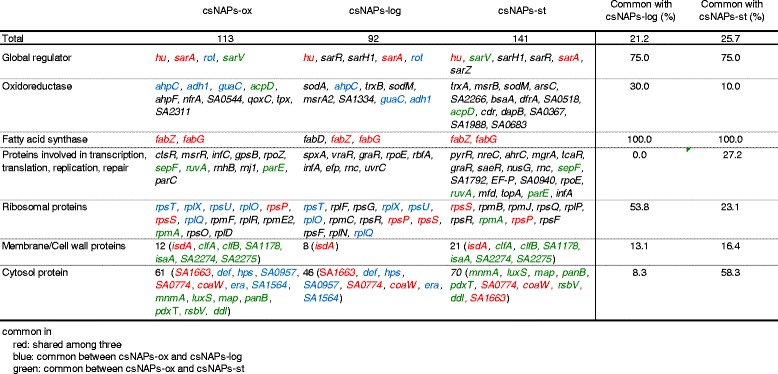
Summary of csNAPs-ox, csNAPs-log, and csNAPs-st

## Discussion

Previous analyses of NAPs in *E. coli*, *P. aeruginosa*, *B. subtilis*, and *S. aureus* in the log and stationary phases revealed that bacterial nucleoid contains global regulators, oxidoreductases, and fatty acid enzymes both in the log and stationary phases [27]. In this study, we found the same feature in the csNAPs in *S. aureus* under the oxidative stress (Table 3 and Additional file 1: Table S2), suggesting that clumped nucleoid sustains significant parts of the nucleoid functions under the oxidative stress.

The csNAP list does not allow us to discuss the whole constituents of the nucleoid and even MrgA was not included in the list, but it is useful to know factors that exist in the nucleoid [27]. However, csNAPs with low emPAI values might require careful confirmation on their subcellular localizations. For example, IsdA, which is one of the surface receptor components of the Isd system [33], was identified as common csNAPs with low emPAI values among the three conditions (0.1 in log phase, 0.2 in oxidative stress). Whether IsdA is the *bona fide* component of the nucleoid has not been tested.

We consider that it is safe to regard proteins with high emPAI values as the genuine nucleoid components. The major csNAPs with top 10 highest emPAI values were listed in Table 4. Again, ribosomal proteins dominated major parts of the list, and it was prominent in csNAPs-ox. This is in consistence with the fact that the nucleoid clumping by artificial expression of MrgA as well as by the endogeneous MrgA expression by oxidative stress allows cells to sustain proliferation [32], where the gene expression must be active, unlikely to the *E. coli* compacted nucleoid in stationary phase or starved conditions.

**Table 4:**
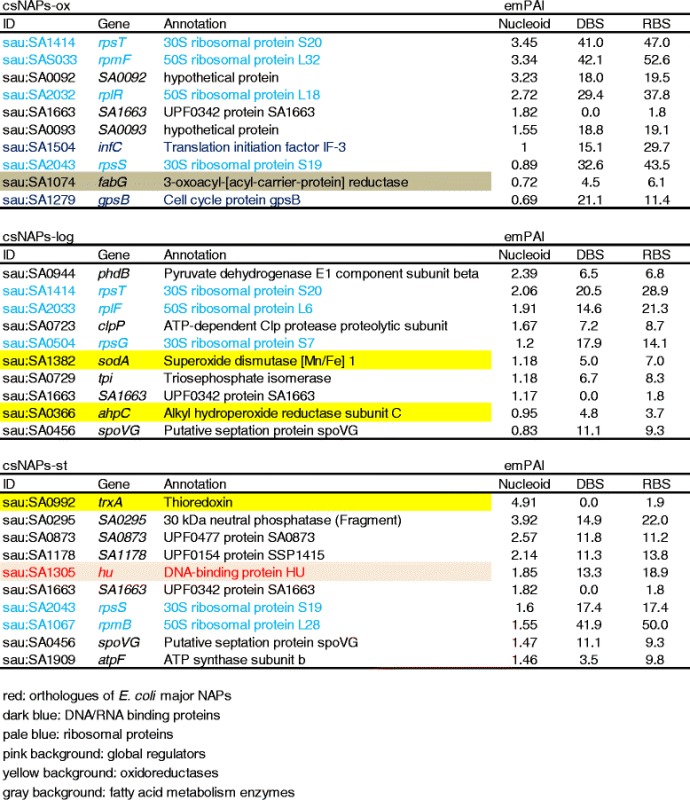
csNAPs-ox, csNAPs-log, and csNAPs-st with top 10 highest emPIA values

The fatty acid enzyme FabG (3-oxoacyl-ACP reductase) is listed in major csNAPs-ox. FabG catalyzes reduction of a 3-oxo-acyl-ACP intermediate during the elongation cycle of fatty acid biosynthesis [34]. Though not included here, the emPAI value of FabZ ([3R] -hydroxymyristoyl-ACP dehydratase) was also significant (0.24). FabZ is (3R)-hydroxymyristoyl-[acyl-carrier-protein] dehydratase involved in fatty acid synthesis [35]. FabG and FabZ have no predictable DNA/RNA binding characteristics. The anchoring mechanism that locates these enzymes on nucleoid is not known, and NAPs that interacted with these enzymes is not reported so far in either *S. aureus* [36] or *E.coli*. It is interesting future work to explore for the interacting factors with FabZ or FabG, which might play key roles in the crosstalk between nucleoid and other cellular functions.

Among the major csNAPs-ox, proteins with unknown function were SA1663, and SA0092/SA0093. The SA0092 and SA0093 are paralogue genes. The DNA/RNA binding prediction score was low in SA1663 (0.0/1.8), suggesting that SA1663 interacts with other nucleoid factors. On the other hand, the DNA/RNA binding prediction scores were high in both SA0092 and SA0093 (18.0/19.5, 18.8/19.1, respectively), suggesting that they might directly interact with nucleic acids. Curiously, the SA0092 and SA0093 were previously identified as “conserved staphylococcal antigens (Csa)” [37]. Some of Csa proteins are thought to be membrane protein or secreted one. The subcellular localization of Csa might be changed in response to the oxidative stress.

## Conclusion

The present study revealed that nucleoid constitutively holds Hu, SarA, FabG, and ribosomal proteins even under the oxidative stress, reflecting the active functions of the clumped nucleoid, unlikely to the dormant *E. coli* nucleoid compacted in the stationary phase or starvation. The NAPs list described here is relevant to study the *S. aureus* physiology under oxidative stress, especially in phagocytic cells in which *S. aureus* can survive and further disseminate to cause severe infectious diseases.

## Methods

### Bacterial growth conditions

*S. aureus* strain N315 was grown as described previously [27]. The glycerol stock of *S. aureus* was inoculated in 10 mL of Brain Heart Infusion (BHI) medium (Difco) and cultured at 37 °C with shaking at 180 rpm (BR-15, TAITEC). Two hundred fifty μl of the overnight culture was inoculated into 25 mL of fresh BHI medium and grown at 37 °C with shaking at 180 rpm until OD_600_ reached at 0.7 (log phase). The stationary phase culture was collected 12 to 14 hours after the inoculation. The culture under oxidative stress was collected 30 min after the addition of 20 μM (final conc.) 9, 10-phenanthrenequinone (PQ) [38] to the log phase culture. *S. aureus* can grow in the presence of PQ. The growth is transiently delayed by the addition of PQ, but the final yield at the stationary phase is not affected (Additional file 2: Figure S1). The cell density was determined by measuring the absorbance at 600 nm (Gene spec III).

### Preparation of nucleoid and soluble fractions

Nucleoid was isolated as previously described with some modifications [27]. Cells were harvested from 25 mL (log phase and oxidative stress) or 2 mL (stationary phase) cultures by centrifugation at 4 °C, and washed once with ice-cold Buffer A (10 mM Tris-HCl [pH 8.2], 100 mM NaCl, and 20% sucrose). Cells were suspended in 0.5 mL of ice-cold Buffer A followed by the addition of 0.1 mL of ice-cold Buffer B (100 mM Tris-HCl [pH 8.2], 50 mM EDTA, 0.6 mg/mL lysozyme, and 100 μg/mL lysostaphin). The mixture was incubated for 15 min at 25 °C. Then, 0.5 mL of ice-cold Buffer C (10 mM Tris-HCl [pH 8.2], 10 mM EDTA, 10 mM spermidine, 1% Briji-58, and 0.4% deoxycholate) was added, followed by the incubation for 30 min at 25 °C. The lysed cell suspension was loaded onto 10-30% linear sucrose density gradients containing 10 mM Tris-HCl (pH 8.2) and 100 mM NaCl and centrifuged at 10,000 rpm for 50 min at 4 °C (Beckmann SW 40 Ti rotor). The top 750 μl was collected by micro-pipet: top (soluble) fraction. Following fractions were harvested by using ATTO PERISTA pump. To quantify the DNA, fifty μl aliquot from each fraction was mixed with 200 ng/ml (final conc.) DAPI, and the fluorescence was measured (excitation: 350 nm, emission: 460 nm).

### Preparation of envelope fraction

Preparation of the envelope fraction was performed as previously described [27]. Briefly, the cells were harvested and suspended in 0.5 mL ice-cold Buffer A and 0.1 mL ice-cold Buffer B as described above. The mixture was incubated for 5 min at 25 °C, followed by the addition of 1 mM (final conc.) phenylmethylsulfonyl fluoride (PMSF). The lysate was sonicated in ice-cold water bath, and the debris was removed by centrifugation. The supernatant was collected, and 5 μg RNase, 10 U DNase, and 40 mM (final conc.) MgCl_2_ were added. After 60 min incubation at 37 °C, the envelope fraction was collected as pellets by centrifugation at 20,000 ×g for 60 min at 4 °C.

### LC-MS/MS

All analyses were carried out as previously described [27]. Briefly, tryptic digestion of in-gel proteins was performed from the each lane of the Coomassie Brilliant Blue (CBB)-stained SDS-PAGE gels (8.5 cm × 6 cm). Tryptic peptides were extracted by sonication in 50% acetonitrile/0.1% trifluoroacetic (TFA) and the supernatants were collected. Again, the supernatants were collected after extraction by sonication in 75% acetonitrile/0.1% TFA. The samples were dried by the MicroVac (Tomy Digital Biology, Tokyo, Japan) and suspended in 2% acetonitrile/0.1% TFA, then further analyzed by LC-MS/MS [27]. Data analysis was performed using a Mascot Server (Matrix Science). Raw data were processed by the SwissProt bacteria subset database (Release 57.4, June 16, 2009) with search parameters as described [27]. The criteria of positive identification were as follows: identification of at least 2 peptides with more than 7 amino acids, and a significant threshold of *P* < 0.05. LC-MS/MS analysis was not repeated because enough number of peptides was detected.

### Selection of csNAPs

We selected “contaminant-subtracted NAPs (csNAPs)” as described in previous study [27]:

csNPAs = “Proteins detected only in the nucleoid fraction” + “Proteins calculated to be relatively abundant in the nucleoid fraction.”

- “Proteins detected only in the nucleoid fraction”: Proteins detected only in the nucleoid fraction in a given condition.
- “Proteins calculated to be relatively abundant in the nucleoid fraction”: The number of peptides detected by LC-MS/MS is a good benchmark to estimate the quantity of proteins. If the number of peptides of a certain protein identified in the nucleoid fraction is larger than that of the other fractions, the protein is thought to be abundant in the nucleoid. The total number of peptides detected by the LC-MS/MS was used to normalize the data, because it reflects the whole protein quantity. We selected proteins with a ratio higher than 3 as csNAPs.

### Prediction of DNA/RNA binding abilities

The DNA/RNA binding sites of the csNAPs were predicted as described previously [27] using BindN+ (http://bioinfo.ggc.org/bindn+/) [39]. Briefly, we set the criterion for the search as ‘the predicted DNA/RNA binding residues with expected specificity equal to 90%’, and then estimated the percentages of DNA/RNA binding residues in a protein. The proteins having high DNA/RNA binding ability were set as over 10%, which was based on the description in previous study [27].

### Western blot analysis

To prepare the whole cell lysate for Western blot analysis, cells were harvested from 1 mL (log phase and oxidative stress), or 100 μL (stationary phase) culture, and washed once with ice-cold PBS (pH 7.4). Cells suspended in 250 μL PBS (pH 7.4) were disrupted by the 10μg lysostaphin treatment at 37 °C, followed by the addition of 1 mM (final conc.) PMSF. The lysate was sonicated to destruct the viscous genome DNA. The protein in the whole cell lysate, as well as in each fraction, was quantified by using DC protein assay kit (Bio-Rad).

Western blot analyses using anti-HU IgG [40] or anti-MrgA IgY [24] were carried out as previously described [41]. Goat anti-rabbit IgG and goat anti-chicken IgY conjugated with alkaline phosphatase (Promega) were used as second antibodies.

## Additional files

Additional file 1: Table S1. NAPs of oxidative stress condition (20 μM PQ, 30 min), **Table S2.** csNAPs of oxidative stress condition (20 μM PQ, 30 min): csNAPs-ox, **Table S3.** Ribosomal proteins detected in the nucleoid fraction. (XLSX 50 kb)

Additional file 2: Figure S1. Growth curves of *S. aureus* N315 in normal condition (BHI) and in oxidative stress (BHI + PQ). Cells were grown in BHI medium at 37°C with shaking at 180 rpm. 20 μM PQ was added at the log phase (shown by arrow). (DOCX 28 kb)
