# Supplementary figures and images for: Identification of nucleoid associated proteins (NAPs) under oxidative stress in Staphylococcus aureus
Source: BMC Microbiol. 2017 Oct 2;17:207. doi: 10.1186/s12866-017-1114-3 (PMC5625760; doi:10.1186/s12866-017-1114-3)

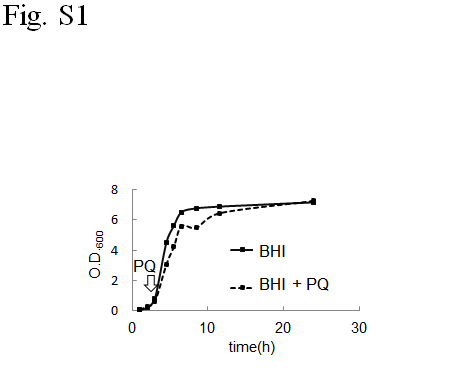

Supplement: Supplementary file 2 — Growth curves of S. aureus N315 in normal condition (BHI) and in oxidative stress (BHI + PQ). Cells were grown in BHI medium at 37°C with shaking at 180 rpm. 20 μM PQ was added at the log phase (shown by arrow). (DOCX 28 kb) [file 12866_2017_1114_MOESM2_ESM.docx]
